# “It cannot depend solely on the parents”—professionals’ perspectives on patient journeys of children with chronic conditions across health and social care in Germany

**DOI:** 10.1007/s00431-026-07533-x

**Published:** 2026-10-22

**Authors:** Carmen J. Herr, Angélique Herrler, Freia De Bock

**Affiliations:** 1 Department of General Paediatrics, Neonatology and Paediatric Cardiology, Medical Faculty and University Hospital Düsseldorf, Heinrich Heine University Düsseldorf, Moorenstraße 5, 40225 Düsseldorf, Germany; 2 Department of Pediatrics, University Medical Center of the Johannes Gutenberg University, Langenbeckstraße 1, 55131 Mainz, Germany; 3 Charité – University Medical Center Berlin, corporate member of Freie Universität Berlin and Humboldt-Universität zu Berlin, Institute for Medical Sociology and Rehabilitation Science, Charitéplatz 1, 10117 Berlin, Germany

**Keywords:** Integrated care, Patient journey, Childhood chronic conditions, Care coordination, Health equity, Health services research

## Abstract

***Purpose*:**

Professionals in healthcare, social services and the education system are confronted with a growing number of children with chronic conditions. Complex treatment plans, care coordination and cross-system care needs result in additional demands on professionals. This study aimed to explore professionals’ perspectives on the patient journeys of children with chronic conditions in Germany and identify opportunities to improve care.

***Methods*:**

This qualitative study used semi-structured interviews conducted from May 2023 to February 2024. A purposive sample of professionals working with children with chronic conditions (attention-deficit/hyperactivity disorder, autism spectrum disorder, developmental disorders, cerebral palsy, bronchial asthma, type 1 diabetes) was recruited from the health, social care, and education systems in Germany. Sampling was guided by an assessment of thematic saturation. Data were analysed using structuring qualitative content analysis.

***Results*:**

Twenty-two professionals participated, including physicians (*n =* 7), therapists (*n =* 8) and social workers and counsellors (*n =* 7). Care was perceived as fragmented, difficult to navigate, and poorly coordinated across health, social care, and education systems. Key barriers included long waiting times, unclear care pathways, and limited interprofessional communication. Greater difficulties in navigating care and support were perceived among families facing socioeconomic disadvantage or language barriers.

***Conclusion*:**

Professionals perceived current care structures as insufficiently meeting the needs of children with chronic conditions, particularly where families face greater barriers to navigating services. Strengthening cross-system coordination and accessibility may improve patient journeys. Future research should examine how barriers vary according to socioeconomic circumstances and across stages of the patient journey and evaluate condition-independent approaches to improving care.

*Trial registration:* This study was registered in the German Clinical Trials Register, ID: DRKS00032139, on 07 November 2023.

**Table:** 

**What is Known:** *• Research on patient journeys of children with chronic conditions has primarily focused on individual, often rare, conditions.* *• Evidence spanning multiple chronic conditions and the healthcare, social services, and education systems remains highly limited.*	
**What is New:** *• Drawing on diverse professionals’ perspectives, this study provides a cross-system, cross-condition view of paediatric patient journeys.* *• It identifies common barriers to care coordination and access across systems and diagnoses, highlighting actionable opportunities for integrated care.*

**Supplementary Information:**

The online version contains supplementary material available at https://doi.org/10.1007/s00431-026-07533-x.

## Introduction

The rising prevalence of chronic conditions in children poses new challenges for professionals involved in their care [1–3]. Management is characterized by complex treatment plans, intensive care coordination and needs extending beyond the healthcare system to social services and education [4–6]. Chronic conditions in childhood are associated with high per-patient costs [7, 8], as well as poorer outcomes for children and greater burden for their caregivers. Compared with children without chronic conditions, affected children have been shown to experience lower health-related quality of life [9], poorer educational outcomes [10] and reduced social participation [11] while their caregivers report greater burden and stress [12–16].

Patient journeys are increasingly used to identify opportunities to improve care [17–20]. The concept of the patient journey can be traced back to the broader concept of the customer journey, which describes an individual’s experience across multiple interactions or “touchpoints” with an organization or service over time [21]. In healthcare, the patient journey concept has been adapted to examine patients’ experiences across encounters and services and to identify opportunities to improve the coordination and quality of care [22, 23]. Importantly, the journey extends beyond individual care encounters to encompass transitions between services and the interactions and processes that shape patients’ experiences across the continuum of care [17, 24].

So far, research on children’s patient journeys has largely focused on rare conditions and remains predominantly confined to the medical system [25–30]. Owing to the rarity of these conditions, many studies focus on condition-specific constraints, such as diagnostic delay [30–33], rather than on shared challenges and structural barriers affecting children across different conditions [29, 31, 32]. Research adopting a cross-condition perspective that extends beyond the medical system remains limited, despite evidence of shared characteristics across conditions and unmet social care needs in this population [34–36].

Existing patient journey research has predominantly captured patients’ experiences, while the perspectives of professionals involved in delivering and coordinating care have received comparatively less attention [18, 37]. While patients’ accounts provide direct insight into their experiences of the journey, professionals’ perspectives can provide complementary insight into the organizational and structural conditions shaping how these journeys unfold across services and systems. Considering both perspectives can therefore provide a more comprehensive understanding of structural and organizational factors that shape children’s and their caregivers’ experiences across healthcare, social services, and education, and consequently the identification of opportunities to improve coordination and continuity of care and support.

This qualitative study contributes to this broader understanding by examining professionals’ perspectives on the factors shaping patient journeys of children with chronic conditions across healthcare, social services, and the education system in Germany.

We adopt a cross-condition, cross-system perspective to identify common challenges and structural factors that extend beyond individual diagnoses and across the above-mentioned system boundaries. In this study, patient journeys refer to the trajectories of children and families through and across healthcare, social services, and education, encompassing their interactions with multiple care settings and actors over time [38]. This systems-oriented conceptualization builds on work by Carayon and colleagues [38] and the subsequent SEIPS 3.0 framework, which conceptualizes patient journeys as distributed across multiple work systems and over time [39]. From this perspective, patient journeys can be understood through different perspectives on the systems and interactions that shape them; SEIPS 3.0 illustrates this systems-oriented approach by describing how a collection of “snapshots” of work systems over time can contribute to an understanding of the patient journey [39]. While patients’ and relatives’ perspectives provide direct insight into their experiences of this journey, professionals’ perspectives provide complementary insight into the organizational and structural factors shaping how these journeys unfold. This study extends previous work from our group examining children’s and caregivers’ direct experiences of these patient journeys [36] by adding the perspectives of professionals involved in providing and coordinating care. The analysis in this paper focuses on challenges in coordinating services across disciplines and sectors, navigating complex care and support systems, and ensuring equitable access to appropriate care and support.

## Methods

### Study design and setting

This qualitative study examined professionals’ perspectives on children’s patient journeys. Semi-structured interviews [40] were conducted, transcribed verbatim and analyzed through structuring qualitative content analysis [41].

The study is set in the German health and broader care system. Care for children with chronic conditions operates within a federal structure, with responsibilities shared across the federal level (healthcare), the federal states (education) and municipalities (public health and youth services) [42]. Structures and service provision thus vary across federal states and regions due to differences in local service structures, delivery arrangements and responsible institutions. Although schools fall under state-level responsibility, the support available to children with chronic conditions may also vary at the regional and local levels and depend on local structures and policies. For example, school-based health services are not systematically implemented, with school nurses largely limited to pilot projects [43, 44].

The study was registered in the German Register for Clinical Trials (DRKS00032139) and received ethical approval from the Medical Ethics Review Board of the Medical Faculty of Heinrich-Heine-University Düsseldorf (2022–2290). Written informed consent was obtained by all participants. The COREQ checklist was used to systematically review the reporting of relevant methodological and procedural aspects of the qualitative study [45]. The checklist is provided in the supplementary material.

### Sampling and recruitment

Participants were recruited following a purposive sampling approach [46, 47]. Recruitment was conducted through specialist societies (e.g. Berufsverband der Kinder- & Jugendärzte (BVKJ) e.V.) and interdisciplinary professional associations, such as the Westdeutsches Zentrum für Kinder- und Jugendgesundheit (WZKJ)). Additional participants were recruited through social paediatric centres, children’s hospitals, regional healthcare stakeholder meetings in several German federal states and family advisory centres in North Rhine–Westphalia. Recruitment included professionals from different regions in several federal states to capture variation in local and regional health-related service contexts. Sampling was not stratified by federal state. As recruitment was conducted through these channels rather than from a defined sampling frame, the number of professionals reached and reasons for non-participation could not be systematically recorded. To enable the identification of common challenges across different chronic conditions, the project purposively included conditions that vary in clinical characteristics, complexity, care needs and the extent to which children and families interact with healthcare, social services and the education system. The selected conditions comprised common chronic somatic and mental or behavioural conditions, as well as developmental and neurological conditions, thereby capturing a broad range of patient journeys and support needs. Eligible participants were professionals from the medical, social services and education system, practising in Germany and regularly working with children with one or more of the following diagnoses: behavioural and emotional disorders with onset usually occurring in childhood and adolescence (ICD-10: F90–94), bronchial asthma (ICD-10: J45), type 1 diabetes mellitus (ICD-10 E10), developmental disorders (ICD-10: F80–84) and cerebral palsy (ICD-10: G80). Professionals had to be able to participate in a 45–60-min interview in German. For reasons of data protection, employees of University Hospital Düsseldorf (our hospital) were not included in the study. Estimated sample size was set at 15 participants, based on empirical recommendations from previous studies [48, 49], but was allowed to be driven by data saturation [48].

### Design and development of the interview guide

The development of the interview guide was initiated by a literature review, followed by a collection of a preliminary set of questions. Questions were discussed with professionals from different fields (medicine, health sciences, law, health economics, health insurance, epidemiology), then sorted and subsumed under open-ended narrative prompts [50]. Two pilot interviews were conducted with a physician and a counsellor to assess question clarity and comprehensibility, followed by a revision of the interview guide. Table 1 provides an overview of topics addressed in the semi-structured interview guide. The full interview guide is provided in the supplementary material.

**Table 1:** Topics addressed in the interview guide

Interview guide
*Professional experience, services provided*
- Occupation, professional experience - Description of typical health condition(s) in whose treatment or care professional is involved
*Experiences with and insights into patient journeys*
- Typical course of a patient journey of a child with a chronic condition from first symptoms to diagnosis and treatment - Temporal sequence of elements in patient journeys, care network, referrals, interdisciplinary exchange, waiting times, social participation of children, accessibility of care
*Positive aspects of service provision*
- Key elements for successful service provision - Support needed to sustain or expand these well-functioning structures
*Challenges in service provision*
- Areas of improvement and major challenges in service provision - Additional efforts or costs for professionals associated with these challenges - Resulting burdens on children with chronic conditions and their families
*Recommendations on indicators of patient journey quality*
- Ideas on indicators of quality of care provided in the patient journey
*Wishes, suggestions for improvement of care, priorities*
- Course of an ideal patient journey of a child with a chronic condition
- Other topics of concern not yet addressed in the interview

### Data collection and analysis

Data collection ran from May 2023 until February 2024. Healthcare professionals were interviewed via telephone to enable a broader geographical coverage across Germany. Participants received a voucher as expense allowance. Interviews were conducted by CJH (female, physician, medical anthropologist) and AH (female, health scientist). Participants had an introductory telephone call with the researchers before the interview. Interviews were audio-recorded, transcribed verbatim and pseudonymized.

Data were analyzed using structuring qualitative content analysis according to Kuckartz and Rädiker [51]. The analysis followed an iterative process combining deductive and inductive approaches. Main categories were initially developed and applied deductively to structure the material. Subcategories were subsequently developed inductively from the data and iteratively refined. The final category system was then used to code the complete dataset, followed by a descriptive and interpretive analysis of main categories and subcategories across interviews and professional groups [51].

This analytical approach was combined with an assessment of thematic saturation to determine the final number of study participants [48, 52, 53]. The New Information Threshold (additional codes identified in a run length compared to the number of codes identified in base size) was set to ≤ 5% [48]. Base size was fixed at six interviews, which were independently coded by AH and CJH. The preliminary categories derived from the material during base size analysis were synthesized in a conceptual map used to create clusters and assess the initial informational scope [54]. A run length of three interviews was independently coded by AH and CJH, aimed at inductively creating subcategories. Newly derived codes were added to the conceptual map, which was revised in each step. After two run lengths, the New Information Threshold was reached. Ten additional interviews were conducted to enhance depth, richness, and representation across different professional groups and conditions [53, 55, 56]. The conceptual map served AH and CJH as the basis for consolidating and labelling main categories and subcategories, and constructing the final codebook.

CJH, assisted by two colleagues, then coded all interviews using the final codebook. Coding of pseudonymized interview transcripts was carried out in MAXQDA® version 24 and reviewed by AH. Following coding of the complete dataset, the coded material was synthesized across main categories and subcategories. Subsequently, CJH conducted an in-depth analysis of the coded material across all interviews, compiling summaries of the findings. These summaries were used to identify commonalities across main categories and subcategories, as well as professional groups [51]. Analytical findings were discussed collaboratively by the research team and then further refined by CJH.

## Results

**Fig. 1:**
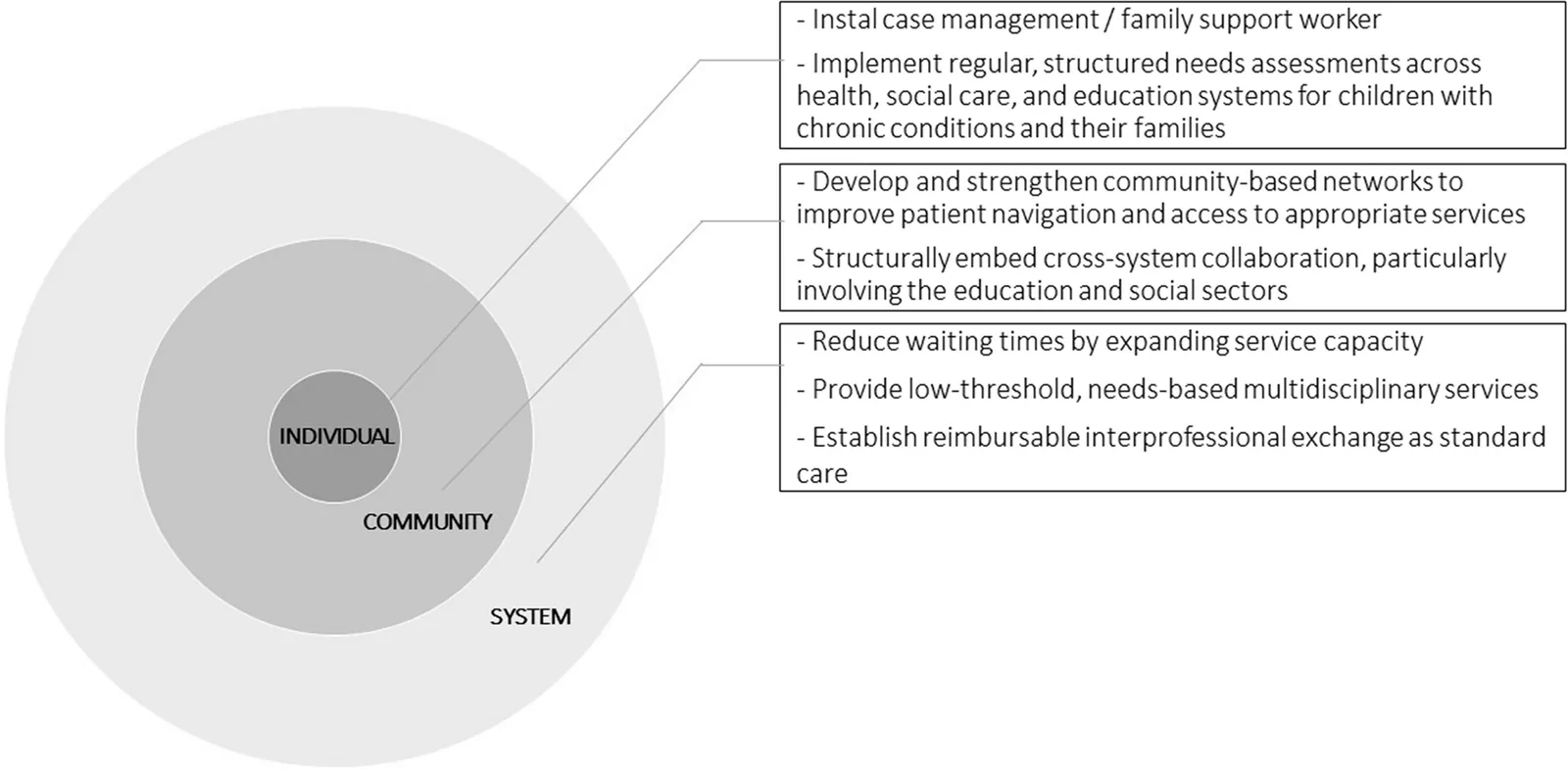
Professionals’ demands and recommendations for improvement of health and social care for children with chronic conditions, synthesized across individual, community, and system levels

The findings are presented as an analytically condensed synthesis of the coded material. The results are structured into three overarching findings: interdisciplinary and interprofessional exchange, navigating a complex and fragmented service landscape, and challenges in service provision. Within these findings, related sub-findings are presented that bring together relevant aspects identified across the more fine-grained coding system. Given its cross-condition perspective, the analysis primarily sought to identify commonalities across professional perspectives, diagnoses, and care systems; differences between these groups were not systematically compared. Where profession- or system-specific perspectives were relevant to understanding a finding, these are reflected in the results. Based on the findings, professionals’ demands and recommendations for improvement are summarized across individual, community, and system levels in Fig. 1. This presentation does not correspond one-to-one to the hierarchical structure of the final codebook, which is provided in the supplementary material.

### Sample

A total of 22 interviews were conducted with professionals in health, social care and education, involved in the care for children with chronic conditions. The distribution of professions over diagnosis groups is shown in Table 2. Participant quotations are presented in English; the original German versions are provided in Supplementary Table 1.

**Table 2:** Sample characteristics

Professional group	Diagnosis group	Number of participants	Gender of participant	Federal state
Occupational therapy	- Behavioural and emotional disorders with onset usually occurring in childhood and adolescence (ICD-10: F90-94) - Developmental disorders (ICD-10: F80-84)	3	Male: 1 Female: 2	North Rhine-Westphalia (3)
Speech therapy	- Behavioural and emotional disorders with onset usually occurring in childhood and adolescence (ICD-10: F90-94) - Developmental disorders (ICD-10: F80-84), - Cerebral palsy (ICD-10: G80)	2	Male: 0 Female: 2	Hamburg (1) Bavaria (1)
Physiotherapy	- developmental disorders (ICD-10: F80-84), - cerebral palsy (ICD-10: G80)	3	Male: 0 Female: 3	North Rhine-Westphalia (3)
Child and adolescent psychology and psychiatry	- Behavioural and emotional disorders with onset usually occurring in childhood and adolescence (ICD-10: F90-94) - Developmental disorders (ICD-10: F80-84) - Type 1 diabetes mellitus (ICD-10: E10)	3	Male: 0 Female: 3	North Rhine-Westphalia (1) Rhineland-Palatinate (2)
Social work, counselling and school social work	- Behavioural and emotional disorders with onset usually occurring in childhood and adolescence (ICD-10: F90-94) - Developmental disorders (ICD-10: F80-84) - Type 1 diabetes mellitus (ICD-10: E10)	7	Male: 1 Female: 6	North Rhine-Westphalia (5) Rhineland-Palatinate (1) Hesse (1)
Paediatrics	- Behavioural and emotional disorders with onset usually occurring in childhood and adolescence (ICD-10: F90-94) - Bronchial asthma (ICD-10: J45) - Type 1 diabetes mellitus (ICD-10: E10) - Developmental disorders (ICD-10: F80-84) - Cerebral palsy (ICD-10: G80)	4	Male: 3 Female: 1	North Rhine-Westphalia (1) Rhineland-Palatinate (2) Bremen (1)

### Interdisciplinary and interprofessional exchange

Participants emphasized the importance of interprofessional and interdisciplinary exchange, which they assessed as “oftentimes unsatisfactory” (P 12 §49; outpatient paediatrician). They highlighted professional exchange and collaboration as essential for high-quality care; however, they reported that, “coordination across sectors or structures is incredibly cumbersome” (P 12 §49; outpatient paediatrician), making it “not possible to establish a common thread for the child” (P 8 §36; counsellor). These difficulties were particularly evident where responsibilities, information, and communication crossed organizational or system boundaries. They criticized the “lack of clear goal-setting” (P 15, §73; physiotherapist) and coordination between professionals, which they felt could lead to ineffective care efforts and poorer therapy outcomes. Professionals perceived high data protection standards in Germany to “hinder communication” (P10, §63; school social worker) and impede short-term exchange between professionals. Formats like round-table meetings, in which professionals from different disciplines and systems (e.g. physicians, social workers, therapists, early childhood educators) discuss a patient’s case, coordinate treatment and supportive measures and establish a treatment plan, were mentioned as positive examples. However, participants criticized that the additional efforts required for treating or counselling children with chronic conditions, including care coordination, communication with other professionals, and referrals, were not structurally recognized as billable services and insufficiently reflected in reimbursement systems. Separately, participants identified difficulties in multidisciplinary exchange across system boundaries and suggested that community-based networking services could facilitate such exchange by increasing awareness of local support, fostering personal connections, and improving referrals for families.

### Navigating a complex and fragmented service landscape

Professionals highlighted complexity and administrative opacity of health and social care systems as challenging for both families and the professionals working within them. In particular, physicians reported a lack of overview of existing support structures and social services for children with chronic conditions, noting that this led to patients being referred between professionals without identifying the appropriate point of contact. One participant described this as: “And then it goes back and forth like a ping-pong ball.” (P 11, §33; child psychiatrist). At the same time, physicians reported substantial time pressure during consultations (“you always feel like you’re on the front line” (P11, §33, child psychiatrist), limiting their ability to address topics beyond what they perceived as “medical” within a single visit. Outpatient paediatricians regarded this as part of their role but expressed a wish for additional support, e.g. through a social worker assigned to their practice. As a potential improvement for families, professionals from different disciplines discussed the role of a case manager or family support worker. They envisioned this role as supporting cross-system communication and navigation, assessing support needs in the home environment, facilitating transitions home after inpatient discharge, and coordinating with schools or early childhood education settings. They emphasized the unique challenges in providing care for children with chronic conditions as it “always requires taking into account their age and stage of life” (P9, §49; paediatric neurologist), and highlighted the benefits of developmentally appropriate, long-term and continuous support for children.

### Challenges for professionals in the provision of care

Professionals identified long waiting times as a critical aspect of children’s patient journeys. Examples mentioned included waiting times for diagnostics and therapy as well as for medical aids and severely disabled person identification cards (e.g. 2 years for occupational therapy, 3 years for autism-specific therapy, 1 year for child psychiatry and psychotherapy).

Professionals attributed this to a lack of qualified staff in the healthcare and social care system and raised concerns about delays in diagnosis and therapy “posing a risk to the children’s development” (P 12, §35; outpatient paediatrician). Accordingly, they advocated for “a legal right for these children” (P 8, §50; counsellor) to timely access to healthcare and support services. Otherwise, professionals feared “that some children will not receive the help they need when they need it. And that this will have long-term consequences” (P 19, §55; speech therapist).

Participants highlighted restrictions on children’s social participation, exclusion at school and during leisure activities, and substantial parental stress related to coordination, paperwork and additional care responsibilities. However, they reported that limited time and resources constrained their ability to alleviate the burden on families to the extent they would have liked. Professionals emphasized that these challenges could be particularly pronounced for families facing language barriers or limited system navigation skills. They described the quality and accessibility of support as being influenced by parents’ abilities and resources, criticizing that: “It cannot depend solely on the parents” (P8, §40; counsellor). Professionals across different professional groups further highlighted how families with fewer resources may face particular difficulties in advocating for their children and navigating complex administrative structures. As one participant noted, parents with greater educational and financial resources “often have other options […] to navigate the bureaucratic jungle,” while some families “do not have this luxury” (P13, §99; school social worker).

A positive example mentioned by professionals was early intervention (“Frühförderung”), a specialized support service for children aged 0–6 years, funded by regional authorities and health insurance providers. Professionals perceived the service as “very, very low threshold” (P5, §59; outpatient paediatrician), needs-based and multidisciplinary, independent of a confirmed diagnosis and characterized by short waiting times.

## Discussion

The findings of this study investigating professionals’ perspectives on patient journeys of children with chronic conditions in Germany suggest that care is widely perceived as unsatisfactory and potentially associated with adverse consequences for children’s development. Professionals highlighted limited interdisciplinary and interprofessional exchange, contributing to fragmented, ineffective care trajectories. Care provision was described as highly complex, with limited awareness of available services and appropriate points of contact among professionals. These structural challenges were perceived to be particularly difficult to navigate for families facing language barriers or socioeconomic disadvantage. Furthermore, professionals emphasized that care for children with chronic conditions required substantial additional effort from both caregivers and professionals, which is not adequately reflected in current reimbursement structures. The patient-journey perspective allowed us to examine how organizational and coordination challenges extend across multiple points of contact and transitions between services, rather than within individual encounters alone. Although professionals typically had insight into only parts of individual journeys, their accounts provided complementary “snapshots” of the structures and processes shaping these trajectories across systems [39].

Our findings suggest that, from professionals’ perspectives, families with fewer educational, financial, or other resources may have fewer opportunities to compensate for gaps in service provision and navigate complex systems, which is particularly concerning as it may exacerbate existing health inequalities in Germany [57]. This is a health equity issue as it impedes children’s right to “access to […] health care services” and the “highest attainable standard of health” set out in article 24 of the United Nations Convention on the Rights of the Child [58]. Moreover, missed opportunities for early intervention, treatment and prevention of comorbidities may contribute to the persistence of high-cost patient trajectories over the life course [59, 60], with potentially greater consequences for children whose families have fewer resources to navigate existing service structures [61].

Our findings suggest that the challenges identified may require complementary responses at different levels, ranging from organizational interventions such as case management to broader efforts to strengthen coordination across services and systems [62], with particular attention to families facing greater barriers to navigating services and accessing support. However, case management and navigator models should not be considered straightforward solutions; evidence from German Innovation Fund projects indicates that their implementation and transfer into routine care can be hampered by challenges in implementation, target-group selection, and evaluation [63]. Early childhood intervention, mentioned by participants as a positive example, has been shown to improve child development and caregiver health outcomes among children with a range of conditions and psychosocial risk factors [64–66]. Programmes built on family-centredness, interdisciplinarity and networking—key pillars of early childhood intervention [64]—may also benefit children with chronic conditions beyond the programme’s target age of 6 years and align with broader integrated care approaches, such as the World Health Organization (WHO) framework on integrated people-centred health services [67].

A core element mentioned in the WHO framework is the coordination of services within and across systems, fostering collaboration across health, social services and the education system [67]. The need for such cross-system integration is also emphasized in the literature on integrated care for children with complex care needs, which highlights the limitations of predominantly single-disease approaches and the importance of integrating healthcare with social and educational services [68]. Integrated care models for children that have been evaluated in the literature vary in composition, outcome measures and evaluation results [69]. Interventions typically combine elements such as case management and needs assessments [70–72]. What is lacking in many models is integration with and strengthening of community-based networks and resources, which studies on social prescribing have identified as a promising approach to bridging gaps between health, social and community services and addressing nonmedical health-related needs [73]. As our study points to the need for a broader, cross-system approach to care, considering the medical needs of the child alongside the psychosocial needs of the child and family, this element seems like a viable approach to providing low-threshold access to care, particularly for families who face greater barriers to navigating fragmented service structures [74].

Another aspect identified by professionals in this study was the impact of long waiting times on children’s patient journeys. Professionals attributed waiting times for diagnostics, therapy and medical aids to both limited coordination and a shortage of qualified professionals. Improved coordination may help reduce delays arising from fragmented referral processes and transitions between services, while capacity constraints may require additional measures to increase the availability of services. Reducing waiting times may therefore require both improved coordination and sufficient service capacity.

Several structural challenges recurred across professional groups and patient populations. Because identifying commonalities across conditions was the primary aim of the cross-condition approach, this recurrence suggests that some opportunities for improving care may lie beyond diagnosis-specific approaches. Given the diversity of chronic conditions in childhood, investing in cross-condition approaches may offer a broadly applicable basis for addressing shared organizational and system-level barriers—such as fragmented services, limited coordination, and difficulties navigating support structures—that are not specific to individual diagnoses. If integrated care models improve patient journeys, they could be scaled to all children with chronic conditions within a region, potentially providing a diagnosis-independent structural layer of care alongside condition-specific services.

### Limitations and directions for future research

To our knowledge, this is the first study to investigate professionals’ perspectives on children’s patient journeys across the medical, social care, and education systems. We were able to highlight difficulties extending beyond system boundaries and identify gaps in communication, coordination, and collaboration across sectors and professional groups, thereby identifying leverage points for improvement of care. However, there are limitations to this study. First, the study was conducted within a specific healthcare system and policy context, which may limit the transferability of the findings; nevertheless, with respect to healthcare system and cross-sectoral fragmentation, many countries face broadly similar structural challenges [75, 76]. Second, although participants were recruited from different federal states and a variety of regional and local contexts, North Rhine-Westphalia was overrepresented in the sample, reflecting the location of several recruitment channels and accessible professional networks. As a qualitative study, the research was not designed to provide a systematic geographical comparison. Healthcare, social services and education structures may vary both between and within federal states due to differences in local service availability, administrative structures, geographic and demographic characteristics, and state-specific regulations. Specific local and organizational contexts that differ from those represented in our sample may not be fully reflected in the findings. Third, the sample was predominantly female, with a more balanced gender distribution only among physicians. This imbalance should be considered when interpreting the findings. However, it reflects the gender composition of the professional groups represented in the study and is broadly consistent with persistent occupational segregation in Germany, where women are particularly concentrated in health and social work, education, and other care-related occupations [77, 78]. Fourth, data on the chronological sequencing of patient journeys was limited, as professionals often only had partial insights into individual care trajectories and were rarely able to specify temporal sequences beyond aspects such as waiting times (highlighting the present lack of cross-system care coordination). The findings should therefore not be interpreted as complete reconstructions of individual patient journeys, but rather as professionals’ perspectives on the systemic structures, processes, and barriers shaping these journeys. Within the education system, perspectives were primarily provided by school social workers; teachers and early childhood educators were not included. Moreover, this study captures professionals’ perspectives and therefore does not directly reflect the experiences and perspectives of children and their families. Children’s and caregivers’ perspectives were examined separately in a related study [36]. The present findings should therefore be understood as a complementary professional and systemic perspective rather than a comprehensive account of children’s and families’ experiences. For example, while parents in our related study described insufficient guidance from physicians on navigating available services, professionals reported that the complexity and fragmentation of existing structures made it difficult for them to identify appropriate points of contact for particular social care needs [36]. Together, these perspectives illustrate how families’ experiences may reflect broader structural barriers to service navigation and coordination. Finally, a selection bias may have occurred, whereby professionals who are dissatisfied with the standard of care for children were more likely to participate in the study. Future research should examine how patient journeys vary according to socioeconomic circumstances, particularly in access to and navigation of care and support services. Longitudinal research at different stages of the patient journey, such as during the first year after diagnosis, could further capture temporal variation in experiences and barriers. Research should also evaluate whether integrated approaches addressing shared structural challenges across conditions can provide a diagnosis-independent approach to improving care.

## Conclusion

Professionals perceived current care structures as insufficiently meeting the needs of children with chronic conditions. Fragmented services, limited interdisciplinary and interprofessional exchange, complex navigation, and insufficient coordination across healthcare, social services, and education were identified as shared structural challenges across conditions, with potentially greater consequences for families facing greater barriers to navigating services. Strengthening cross-system coordination and accessibility may improve patient journeys, while further research should clarify how these challenges differ according to families’ socioeconomic circumstances and across stages of the patient journey, and evaluate condition-independent approaches to improving care.

## Supplementary Information

Below is the link to the electronic supplementary material.

(DOCX 52.6 KB)

(DOCX 53.1 KB)

(DOCX 39.4 KB)

(DOCX 29.2 KB)

(DOCX 23.0 KB)

## Data Availability

The datasets used and/or analysed during the current study are available from the corresponding author on reasonable request. The original participant quotations in German alongside their English translations, the codebook, the full interview guide and the COREQ checklist are provided as supplementary materials.
