# Supplementary material for: “It cannot depend solely on the parents”—professionals’ perspectives on patient journeys of children with chronic conditions across health and social care in Germany
Source: Eur J Pediatr. 2026 Oct 22;185(11):872. doi: 10.1007/s00431-026-07533-x (PMC13687312; doi:10.1007/s00431-026-07533-x)
Supplement: Supplementary file 1 [file 431_2026_7533_MOESM1_ESM.docx]

**Codebook – English Translation**

*English translation of the German codebook; original code numbering retained.*

|  | **Code** |
| --- | --- |
| **1** | **Other** |
| 1.1 | Effects of COVID-19 |
| 1.2 | Side course / disruption |
| 1.3 | No further additions (at this point) |
| **2** | **Application for and approval of services/benefits** |
| 2.1 | No insight into application procedures or approval practices |
| 2.2 | Funding by municipality |
| 2.3 | Funding through other support schemes |
| 2.4 | Funding through integration assistance |
| 2.5 | Funding through care allowance |
| 2.6 | (Unpaid) additional workload for professionals due to rejected applications |
| 2.7 | Service/benefit cannot be billed to statutory health insurance |
| 2.8 | Complex and opaque application processes |
| 2.9 | Rejected applications |
| 2.10 | Language barriers complicate applications for services/benefits |
| 2.11 | (Lack of) support with applications for services/benefits |
| 2.12 | Particular difficulty: approval of home care needs |
| 2.13 | (Unnecessary) time limits on services/benefits |
| 2.14 | Application based on a provisional or suspected diagnosis |
| 2.15 | Requirement to submit an application |
| **3** | **Professional's area of work** |
| 3.1 | Insight into the typical course of a patient journey |
| 3.2 | Exchange with others: parents |
| 3.3 | Description of one's own professional activities |
| 3.4 | Catchment area |
| 3.5 | Information on professional background |
| 3.6 | Creating a long-term (future and vocational) perspective |
| 3.7 | Own continuing education and training |
| 3.8 | Strengthening everyday living skills |
| 3.9 | Professional connects affected individuals with others/services |
| 3.10 | Professional guides others / makes onward referrals |
| 3.11 | Intermediary role, coordination |
| 3.12 | “Translating” between parents and child |
| 3.13 | Professional performs tasks beyond the actual scope of responsibility |
| 3.14 | Degree to which children are involved in decisions by the professional |
| 3.15 | Individual adaptation of therapy goals to and with the child |
| 3.16 | Education and explanations about the condition |
| 3.17 | Collaboration with other professionals |
| 3.18 | Exchange with others: school and daycare |
| 3.19 | Exchange with others: specialists and therapists |
| 3.20 | Exchange with public offices and authorities |
| 3.21 | Reasons/conditions for using the professional's services |
| 3.22 | Training for caregivers/support staff on the condition and treatment management |
| 3.23 | Issuing referrals and prescriptions/orders |
| 3.24 | Bureaucracy, administrative tasks |
| 3.25 | Psychosocial support for families |
| 3.26 | Support with applications for services, access, and general assistance |
| 3.27 | Child protection |
| 3.28 | Accompanying families to appointments, home visits |
| **4** | **Contact with conditions** |
| 4.1 | Developmental disorders in general (excl. Autism) |
| 4.2 | Motor developmental disorder |
| 4.3 | Cerebral palsy |
| 4.4 | Diabetes |
| 4.5 | Autism |
| 4.6 | Asthma |
| 4.7 | ADHD |
| **5** | **Routes/referrals to the professional** |
| 5.1 | Parental engagement = faster access |
| 5.2 | Direct referral/mediation by third parties |
| 5.3 | Public offices and authorities |
| 5.4 | Personal recommendation, word of mouth |
| 5.5 | As part of the in-house process/standard |
| 5.6 | During a hospital stay |
| 5.7 | Paediatric practice |
| 5.8 | Daycare, teaching staff, school |
| 5.9 | Other professional |
| 5.10 | Parents' own initiatives |
| 5.11 | Professional notices a clinical sign the child and takes action |
| 5.12 | Parents' internet searches, social media |
| 5.13 | Child's own initiative |
| **6** | **Professional's information about services and points of contact** |
| 6.1 | Networking initiatives by working groups |
| 6.2 | Request: information for paediatric practices about health insurance programmes and support services |
| 6.3 | Consultation with other professionals and points of contact |
| 6.4 | Self-acquired information, own research |
| 6.5 | Through experiences of other patients |
| 6.6 | Through counselling services |
| 6.7 | Central municipal coordination office |
| 6.8 | Self-created opportunities for exchange and contacts |
| 6.9 | Own experience and local network building |
| **7** | **Other points of contact with which the child is in contact** |
| 7.1 | Outpatient follow-up appointments |
| 7.2 | Office for Migration and Integration |
| 7.3 | Autism therapy centre |
| 7.4 | Ophthalmologist |
| 7.5 | Counselling services |
| 7.6 | Guardianship authority |
| 7.7 | Diabetes outpatient clinic |
| 7.8 | Occupational therapy |
| 7.9 | Early intervention |
| 7.10 | Early help services |
| 7.11 | Public health department |
| 7.12 | Special education / therapeutic pedagogy |
| 7.13 | Medical equipment/supply provider |
| 7.14 | Ear-Nose-Throat doctors |
| 7.15 | Inclusion aide in daycare or school |
| 7.16 | Youth welfare office |
| 7.17 | Paediatric cardiology |
| 7.18 | Paediatric orthopedics |
| 7.19 | Child and adolescent psychiatry |
| 7.20 | Paediatric practice |
| 7.21 | Speech and language therapy |
| 7.22 | Medication management |
| 7.23 | Neuropaediatrics |
| 7.24 | Physiotherapy |
| 7.25 | Psychology |
| 7.26 | Other therapies |
| 7.27 | Self-help groups |
| 7.28 | Social services |
| 7.29 | Social paediatric centre (SPZ) |
| 7.30 | Social welfare office |
| 7.31 | Specialised centres |
| 7.32 | Treatment/therapy education |
| **8** | **Patient Journey – timelines** |
| 8.1 | Waiting time until service/benefit approval |
| 8.2 | Time until diagnosis |
| 8.3 | Frequency and points of contact with the professional during the patient journey |
| 8.4 | Waiting time for diagnostic appointments |
| 8.5 | Waiting time for therapy appointments |
| 8.6 | Time until access to support services |
| **9** | **Well-functioning aspects of care** |
| 9.1 | Good care structures |
| 9.2 | Flexibility in designing support for families |
| 9.3 | Staffing ratios |
| 9.4 | Appropriate waiting times, rapid access to support |
| 9.5 | No problems with coverage of services/benefits |
| 9.6 | Prescription of medication |
| 9.7 | Good counselling services |
| 9.8 | Parents cope well psychologically with the situation |
| 9.9 | Child copes well psychologically with the situation |
| 9.10 | Collaboration with families |
| 9.11 | Duplicate examinations are not an issue |
| 9.12 | Dealing with foreign languages |
| 9.13 | Interdisciplinary collaboration, communication |
| 9.14 | Coordination and referral |
| 9.15 | Resource-rich parents manage to navigate the system better |
| 9.16 | Well-informed and treating professional |
| 9.17 | Good level of information among families |
| 9.18 | Strong personal commitment and effort by professionals to provide good care |
| **10** | **Facilitators of good care** |
| 10.1 | More/sufficient time |
| 10.2 | Error culture, working climate, leadership style |
| 10.3 | (More) interdisciplinary exchange |
| 10.4 | More professionals needed to maintain and expand good care structures |
| 10.5 | Decoupling services/benefits from diagnosis |
| 10.6 | Early recognition of parents' concerns |
| 10.7 | Rapid access to institutions/services |
| 10.8 | No ideas |
| **11** | **Patient Journey – problem areas** |
| 11.1 | Misallocation of funds and resources |
| 11.2 | Professionals need more time to care for younger children |
| 11.3 | Additional efforts due to coordination |
| 11.4 | Complex care needs: high workload for professionals |
| 11.5 | No healthy food during inpatient stays |
| 11.6 | Little flexibility in designing support for families |
| 11.7 | Difficult interdisciplinary exchange, dependent on individual commitment |
| 11.8 | Too little work with parents when therapy is provided in school/daycare |
| 11.9 | Diagnostic and therapeutic procedures lacking scientific foundation |
| 11.10 | Lack of knowledge in authorities and schools about the needs of children with chronic conditions |
| 11.11 | Importance of diagnosis, including with regard to access to services/benefits |
| 11.12 | Acute needs can only be addressed by professionals through child-protection concerns or similar mechanisms |
| 11.13 | Effects of COVID-19: increased need |
| 11.14 | Challenge: transition to adult medicine |
| 11.15 | Too few support services for the condition |
| 11.16 | (Lack of) information, (knowledge of) counselling services, referral |
| 11.17 | Too few respite/support services for families (psychosocial, etc.) |
| 11.18 | System: unclear access, responsibilities, and entitlement |
| 11.19 | Poorer/more difficult care for families with a migration or refugee history |
| 11.20 | Need for targeted professional support in the home setting |
| 11.21 | Statements by professionals carry different weight (e.g., daycare versus physician) |
| 11.22 | Lack of a (qualified) counselling/general support professional |
| **12** | **Consequences of a suboptimal patient journey** |
| 12.1 | Missed developmental opportunities |
| 12.2 | Psychological burden on professionals |
| 12.3 | Acute health risk |
| 12.4 | Preventable comorbidities |
| 12.5 | Risk to child welfare |
| 12.6 | Psychological difficulties and disorders |
| 12.7 | Development of health-damaging behaviours |
| 12.8 | Poorer well-being, bullying |
| 12.9 | Socially problematic behaviour |
| 12.10 | Child is misunderstood |
| 12.11 | Intrusion into family life |
| 12.12 | Undignified living conditions |
| 12.13 | Constrained, restricted living space |
| 12.14 | Difficulties managing everyday life |
| 12.15 | Worse educational and life-course trajectory |
| **13** | **Desired points of contact for families** |
| 13.1 | New wish/proposal: desired points of contact |
| 13.2 | Consultation with physician including a further plan and points of contact |
| 13.3 | Central, officially organized point of contact/counselling service |
| 13.4 | Paediatric practice desired as central point of contact |
| 13.5 | Referral to other affected individuals/families |
| 13.6 | More information, counselling, and referral support from health insurers |
| **14** | **Ideas for solutions, wishes** |
| 14.1 | New wish/proposal: ideas for solutions, wishes |
| 14.2 | Social workers in paediatric practices |
| 14.3 | Expansion of digital services |
| 14.4 | Information services for professionals on services/benefits and responsibilities |
| 14.5 | More individualised therapy, tailored to the child |
| 14.6 | Location-independent online services reimbursable by health insurance |
| 14.7 | More outpatient rather than fully inpatient services |
| 14.8 | Reduction of waiting times through various measures |
| 14.9 | Education of professionals about conditions |
| 14.10 | Public education about conditions |
| 14.11 | Continuing education and up-to-date professional knowledge |
| 14.12 | Expansion of structures for children with chronic conditions |
| 14.13 | Outreach support services |
| 14.14 | Dedicated support person for the family to provide relief |
| 14.15 | Tiered support services for families experiencing difficulties |
| 14.16 | Peer exchange opportunities for families with a preventive approach |
| 14.17 | Central information, referral, and care-navigation office for families |
| 14.18 | More (trained) professionals and institutions |
| 14.19 | Better basic funding and service/benefit approval |
| 14.20 | Specialised points of contact |
| **15** | **Measures for evaluating the patient journey** |
| 15.1 | New wish/proposal: measures for evaluation |
| 15.2 | Accessibility of the professional to families, communication |
| 15.3 | Parents' implementation of professional recommendations |
| 15.4 | Continuity of care when changing professionals |
| 15.5 | Approval practices for care services/benefits (timelines, rejections) |
| 15.6 | Time between diagnosis/therapy or recommendation and start of service, waiting time for appointments |
| 15.7 | Condition-specific outcomes and trajectories |
| 15.8 | Integration into the school system |
| 15.9 | Dependent on the family's circumstances, willingness, and preferences |
| 15.10 | ICD code for assessing care by the paediatric practice |
| 15.11 | Professionals’ time expenditure required to care for the patient |
| 15.12 | Parents' and children's knowledge about the condition and care system |
| 15.13 | Number of contacts with professionals |
| 15.14 | Care contact between mandatory appointments |
| 15.15 | Number of different points of contact before diagnosis is established |
| 15.16 | Number of inpatient stays |
| 15.17 | Number of emergency presentations due to the condition |
| 15.18 | Availability of family support workers |
| 15.19 | Amount of parental engagement required for good care |
| 15.20 | Parents feel well supported and adequately cared for |
| 15.21 | Burden or relief for the family |
| 15.22 | Participation, realistically possible (independent) leisure activities |
| **16** | **Potentially avoidable efforts and burdens** |
| 16.1 | High bureaucratic burden |
| 16.2 | Repeated diagnostics and examinations, and reasons for these |
| 16.3 | Potentially unnecessary diagnostic/therapeutic procedures, reasons for these |
| 16.4 | Overlap of services in parallel support programmes |
| 16.5 | Unnecessary rejections and expert assessments |
| 16.6 | (Unnecessary) family preference for specialised treatment |
| 16.7 | Families request potentially unnecessary services or medical aids |
| **17** | **Child's everyday environment: daycare and school** |
| 17.1 | Therapy services in school or daycare |
| 17.2 | Rapid return to regular daily life/school |
| 17.3 | Problems at school/another institution rejects the child |
| 17.4 | Inadequate (including staffing) resources in school/daycare |
| 17.5 | Helpful: more flexible forms of school attendance, e.g. online |
| 17.6 | Exclusion from school |
| 17.7 | Too few inclusion aides, rejected applications |
| 17.8 | (Lack of) knowledge about the condition among childcare workers and teachers |
| 17.9 | Child's difficulties with concentration or behaviour |
| 17.10 | Support needed for treatment management at school |
| 17.11 | Inadequate staffing ratio |
| 17.12 | Differences between federal states: focus on daycare and school structures |
| 17.13 | Description of everyday school life |
| 17.14 | Daycare/school as the child's only living environment outside the home |
| **18** | **Ideal patient journey** |
| 18.1 | New wish/proposal for an ideal patient journey |
| 18.2 | Smooth approval of services/benefits |
| 18.3 | More interdisciplinary exchange, cross-system collaboration |
| 18.4 | Detection of clinical signs and initiation of diagnostic assessment by a professional |
| 18.5 | Central point of contact |
| 18.6 | Rapid (provisional) diagnosis, sufficient capacity of the care system |
| 18.7 | Central/good source of information for families, good communication |
| 18.8 | Strengthening families' self-efficacy |
| 18.9 | Consider families' coping and provide outreach support where needed |
| 18.10 | Regular contact with social counselling or psychological services |
| 18.11 | Rapid and closer connection to therapy and support systems |
| 18.12 | Case manager |
| **19** | **Social stigma and prejudice** |
| 19.1 | (No) lack of social acceptance, education, and adaptation |
| 19.2 | Prejudices and stereotypes |
| 19.3 | Parents are blamed for the child's behaviour |
| 19.4 | Professionals suspect parents of neglecting or abusing the child |
| 19.5 | Diagnosis is questioned |
| 19.6 | Test manuals are insufficiently diverse |
| 19.7 | High awareness, little discomfort/fear in dealing with the condition |
| **20** | **Participation** |
| 20.1 | Not achieved |
| 20.2 | Participation works well |
| 20.3 | Child's independence is restricted |
| 20.4 | Participation is generally unrestricted with good treatment management |
| 20.5 | Participation in class trips and school outings |
| 20.6 | Experience: condition is well controllable |
| 20.7 | (Treatment) goals regarding participation |
| 20.8 | Restricted social contacts |
| 20.9 | Support of the child by peers |
| 20.10 | Child does not want to stand out among peers because of the condition |
| 20.11 | (Lack of) services/benefits supporting participation |
| 20.12 | Severe restriction of the child in everyday life |
| 20.13 | Lack of funding to support participation |
| 20.14 | Child managing treatment independently, effects on participation |
| 20.15 | Lack of information about the condition in leisure settings |
| 20.16 | Programmes/supervisors want more information on the condition and safety measures |
| 20.17 | Safety concerns restrict participation |
| 20.18 | Structures in school/daycare make participation more difficult |
| 20.19 | Concrete suggestions for improving participation |
| 20.20 | Bullying |
| 20.21 | Child copes well with the condition |
| 20.22 | School assistant: safety versus independence of the child |
| 20.23 | Lack of leisure/holiday activities for the child |
| **21** | **Professional availability and financial resources** |
| 21.1 | Lack of time for interdisciplinary exchange and communication |
| 21.2 | Lack of resources to treat according to the latest scientific standards |
| 21.3 | Lack of structures to address everyday care problems |
| 21.4 | Lack of professional time for additional/better treatment |
| 21.5 | No professional specialised in the condition available |
| 21.6 | Discharge after initial diagnosis before parents and child feel confident in treatment management |
| 21.7 | (Lack of) regional care structures |
| 21.8 | Long waiting times for diagnostics and therapy due to too lack of professionals |
| 21.9 | Closure of facilities worsens capacity shortages |
| 21.10 | Cost pressures on health insurers lead to application rejections |
| 21.11 | Good availability of professionals |
| 21.12 | Long waiting times, insufficient capacity |
| 21.13 | Inadequate care due to staff shortages |
| 21.14 | Insufficient capacity in paediatric practices for the needs of children with chronic conditions |
| **22** | **Family burden related to care** |
| 22.1 | Long distances, travel time |
| 22.2 | Importance of parental engagement and resources for good care |
| 22.3 | Support for families needed beyond initial diagnosis |
| 22.4 | Burden from rejected applications and reviews by the Medical Service (Medizinischer Dienst, MD) |
| 22.5 | Burden due to cramped housing conditions |
| 22.6 | Organisational and coordination burden on families |
| 22.7 | Repeated hospital stays due to complications |
| 22.8 | Family burdens lead to poorer treatment management |
| 22.9 | Balancing care work and employment |
| 22.10 | Parents' “correct” assessment of the child's developmental level |
| 22.11 | Time required for treatment management at school |
| 22.12 | Time required to accompany the child on school outings and class trips |
| 22.13 | Financial hardship and difficulties |
| **23** | **Family system** |
| 23.1 | Parents' health burden |
| 23.2 | (Lack of) respite and psychosocial support |
| 23.3 | Psychological burden on the child |
| 23.4 | Psychological burden on families |
| 23.5 | Influence of shame |
| 23.6 | Less severe condition is taken less seriously |
| 23.7 | Multiple reasons for (unsuccessful) adaptation to the situation |
| 23.8 | Management by parents: condition well under control |
| 23.9 | Everyday life: things have to be taken into account |
| 23.10 | Relief through having an established diagnosis |
| 23.11 | (No) difficulties accepting the condition |
| 23.12 | Changes in living circumstances influence how the child's condition is managed |
| 23.13 | Particular burden when a sibling is also unwell |
| 23.14 | Problems in everyday life for the child |
| 23.15 | The child's condition is perceived by parents as a disruptive factor |
| 23.16 | Parental separation or illness of a parent results in loss of a caregiver |
| 23.17 | Mainly one parent takes responsibility for condition management |
| 23.18 | Difficulties experienced by affected individuals due to the condition |
| 23.19 | Negotiation processes and conflicts between parents and child regarding the condition and its management |
| 23.20 | Parents' uncertainty in dealing with the condition and the child |
| 23.21 | Parents' concerns about the course of the condition and life prospects |
