# Supplementary material for: “It cannot depend solely on the parents”—professionals’ perspectives on patient journeys of children with chronic conditions across health and social care in Germany
Source: Eur J Pediatr. 2026 Oct 22;185(11):872. doi: 10.1007/s00431-026-07533-x (PMC13687312; doi:10.1007/s00431-026-07533-x)
Supplement: Supplementary file 2 [file 431_2026_7533_MOESM2_ESM.docx]

**Codebook – Deutsche Originalversion**

|  | **Code** |
| --- | --- |
| **1** | **Sonstiges** |
| 1.1 | Effekte Corona |
| 1.2 | Nebenverlauf/Störung |
| 1.3 | (an dieser Stelle) keine Ergänzungen |
| **2** | **Leistungsbeantragung, Bewilligung** |
| 2.1 | kein Einblick in Antragsverfahren, Bewilligungspraxis |
| 2.2 | Finanzierung durch Kommune |
| 2.3 | Finanzierung über sonstige Hilfen |
| 2.4 | Finanzierung über Eingliederungshilfe |
| 2.5 | Finanzierung über Pflegegeld |
| 2.6 | (unbezahlter) Mehraufwand für Fachkraft durch Ablehnung von Anträgen |
| 2.7 | Leistung nicht über Krankenkasse abrechenbar |
| 2.8 | komplizierte und undurchsichtige Beantragungsprozesse |
| 2.9 | abgelehnte Antragsverfahren |
| 2.10 | Sprachbarriere erschwert Leistungsbeantragung |
| 2.11 | (fehlende) Unterstützung bei Leistungsbeantragung |
| 2.12 | besondere Schwierigkeit: Bewilligung häuslicher Pflegebedarf |
| 2.13 | (unnötige) Befristung von Leistungen |
| 2.14 | Beantragung mit vorläufiger Diagnose/Verdachtsdiagnose |
| 2.15 | Notwendigkeit einer Beantragung |
| **3** | **Arbeitsgebiet der Fachkraft** |
| 3.1 | Einblick in typischen Ablauf einer Patient Journey |
| 3.2 | Austausch mit anderen: Eltern |
| 3.3 | Beschreibung der eigenen beruflichen Tätigkeit |
| 3.4 | Einzugsgebiet |
| 3.5 | Angaben zum beruflichen Hintergrund |
| 3.6 | langfristige (Zukunfts- & berufliche) Perspektive schaffen |
| 3.7 | eigene Fort- & Weiterbildung |
| 3.8 | Alltagskompetenz stärken |
| 3.9 | Fachkraft vernetzt Betroffene |
| 3.10 | Fachkraft leitet andere an, vermittelt weiter |
| 3.11 | Vermittlerrolle, Koordination |
| 3.12 | "Übersetzen" zwischen Eltern & Kind |
| 3.13 | Fachkraft erfüllt mehr Aufgaben als eigentlich in Zuständigkeitsbereich fallen |
| 3.14 | Grad der Einbeziehung von Kindern in Entscheidungen durch Fachkraft |
| 3.15 | individuelle Anpassung Therapieziele an & mit dem Kind |
| 3.16 | Aufklärungsarbeit, Erklärungen zu Krankheitsbild |
| 3.17 | Zusammenarbeit mit anderen Fachkräften |
| 3.18 | Austausch mit anderen: Schule & Kita |
| 3.19 | Austausch mit anderen: Fachärzt*innen, Therapeut*innen |
| 3.20 | Austausch mit Ämtern + Behörden |
| 3.21 | Gründe/Erkrankungen für die Inanspruchnahme der Fachkraft-Leistungen |
| 3.22 | Schulungen für Betreuungspersonen zu Krankheit, Therapie-Management |
| 3.23 | Ausstellen von Überweisungen, Verordnungen |
| 3.24 | Bürokratie, Administratives |
| 3.25 | psychosoziale Unterstützung der Familien |
| 3.26 | Unterstützung Beantragung Leistungen, Zugänge, allgemeinen Hilfen |
| 3.27 | Kinderschutz |
| 3.28 | Begleitung Familien zu Terminen, Hausbesuche |
| **4** | **Kontakt mit Krankheitsbild** |
| 4.1 | Entwicklungsstörungen allgemein |
| 4.2 | motor Entwicklungsstörung |
| 4.3 | Cerebralparese |
| 4.4 | Diabetes |
| 4.5 | Autismus |
| 4.6 | Asthma |
| 4.7 | ADHS |
| **5** | **Zubringer zur Fachkraft** |
| 5.1 | Elternengagement = schnellerer Zugang |
| 5.2 | direkte Zuleitung/Vermittlung durch Dritte |
| 5.3 | Ämter & Behörden |
| 5.4 | persönliche Empfehlung, Mundpropaganda |
| 5.5 | als Teil des Inhouse-Prozesses/Standard |
| 5.6 | im Rahmen von Klinikaufenthalt |
| 5.7 | Kinderarztpraxis |
| 5.8 | Kita, Lehrpersonal, Schule |
| 5.9 | andere Fachkraft |
| 5.10 | Eigeninitiativen Eltern |
| 5.11 | Fachkraft sieht Auffälligkeit bei Kind & wird selbst aktiv |
| 5.12 | Internetrecherche Eltern, Social Media |
| 5.13 | Eigeninitiative des Kindes |
| **6** | **Infos der Fachkraft über Angebote & Anlaufstellen** |
| 6.1 | Vernetzungs - Initiativen von Arbeitskreisen |
| 6.2 | Wunsch: Info an Kinderarztpraxis über Krankenkassen Programme, Hilfs-Angebote |
| 6.3 | Rücksprache mit anderen Fachkräften & Anlaufstellen |
| 6.4 | selbst angeeignet, recherchiert |
| 6.5 | über Erfahrungen von anderen Patient*innen |
| 6.6 | über Beratungsstellen |
| 6.7 | zentrale kommunale Koordinierungsstelle |
| 6.8 | selbst geschaffene Austauschmöglkeiten & Kontakte |
| 6.9 | eigene Erfahrung & lokaler Netzwerkaufbau |
| **7** | **weitere Anlaufstellen, mit denen Kind in Kontakt** |
| 7.1 | ambulante Kontrolltermine |
| 7.2 | Amt für Migration & Integration |
| 7.3 | Autismus-Therapie-Zentrum |
| 7.4 | Augenärzt:in |
| 7.5 | Beratungsstellen |
| 7.6 | Betreuungsbehörde |
| 7.7 | Diabetes-Ambulanz |
| 7.8 | Ergotherapie |
| 7.9 | Frühförderung |
| 7.10 | frühe Hilfen |
| 7.11 | Gesundheitsamt |
| 7.12 | Heilpädagogik |
| 7.13 | Hilfsmittelversorger |
| 7.14 | HNO |
| 7.15 | I-Helfer in Kita, Schule |
| 7.16 | Jugendamt |
| 7.17 | Kinder-Kardiologie |
| 7.18 | Kinderorthopädie |
| 7.19 | Kinder- und Jugendpsychiatrie |
| 7.20 | Kinderarztpraxis |
| 7.21 | Logopädie |
| 7.22 | medikamentöse Versorgung |
| 7.23 | Neuropädiatrie |
| 7.24 | Physiotherapie |
| 7.25 | Psychologie |
| 7.26 | sonstige Therapien |
| 7.27 | Selbsthilfegruppen |
| 7.28 | Sozialdienst |
| 7.29 | SPZ |
| 7.30 | Sozialamt |
| 7.31 | Spezialzentren |
| 7.32 | Therapie-Schulungen |
| **8** | **Patient Journey - Zeitschienen** |
| 8.1 | Wartezeit bis Leistungsbewilligung |
| 8.2 | Zeit bis Diagnosestellung |
| 8.3 | Kontaktfrequenz, -punkt mit der Fachkraft in PJ |
| 8.4 | Wartezeit auf Termine für Diagnostik |
| 8.5 | Wartezeit auf Termine für Therapie |
| 8.6 | Zeit, bis Zugang zu unterstützenden Angeboten |
| **9** | **gut funktionierende Aspekte der Versorgung** |
| 9.1 | gute Versorgungsstrukturen |
| 9.2 | Freiraum in der Ausgestaltung der Unterstützung für Familien |
| 9.3 | Personalschlüssel |
| 9.4 | angemessene Wartezeiten, schnelle Anbindung an Unterstützung |
| 9.5 | keine Probleme mit Leistungsübernahme |
| 9.6 | Verordnung Medikation |
| 9.7 | gute Beratungsstellen |
| 9.8 | Eltern bewältigen Situation psychisch gut |
| 9.9 | Kind bewältigt Situation psychisch gut |
| 9.10 | Zusammenarbeit mit Familien |
| 9.11 | Doppeluntersuchungen kein Thema |
| 9.12 | Umgang mit Fremdsprachen |
| 9.13 | interdisziplinäre Zusammenarbeit, Kommunikation |
| 9.14 | Koordination + Weiterleitung |
| 9.15 | ressourcenstarke Eltern kämpfen sich durch |
| 9.16 | gut informierte & therapierende Fk |
| 9.17 | guter Informationsstand bei Familien |
| 9.18 | großer persönlicher Einsatz, Engagement der Fachkraft für gute Versorgung |
| **10** | **Förderfaktoren einer guten Versorgung** |
| 10.1 | mehr/ausreichend Zeit |
| 10.2 | Fehlerkultur, Arbeitsklima, Führungsstil |
| 10.3 | (mehr) interdisziplinärer Austausch |
| 10.4 | mehr Fachkräfte nötig für Erhaltung, Ausbau guter Versorgungsstruktur |
| 10.5 | Entkoppelung Leistung von Diagnose |
| 10.6 | frühzeitiges Erkennen von Sorgen bei den Eltern |
| 10.7 | schnelle Anbindung an Institutionen |
| 10.8 | keine Ideen |
| **11** | **Patient Journey - Problemfelder** |
| 11.1 | Fehl-Allokation von Geldern, Ressourcen |
| 11.2 | höherer Zeitaufwand für Versorgung jüngerer Kinder |
| 11.3 | Mehraufwand durch Koordination |
| 11.4 | Komplexe Versorgungsbedarfe: hoher Aufwand für Fachkraft |
| 11.5 | kein gesundes Essen bei stationärem Aufenthalt |
| 11.6 | kaum Freiheit in der Ausgestaltung der Unterstützung für Familien |
| 11.7 | erschwerter interdisziplinärer Austausch, abhängig von Engagement Einzelner |
| 11.8 | bei Therapie in Schule, Kita zu wenig Elternarbeit |
| 11.9 | Verfahren in Diagnostik, Therapie, fehlende wissenschaftliche Fundierung |
| 11.10 | fehlende Kenntnisse bei Amt, Schule zu Bedarfen chronisch kranker Kinder |
| 11.11 | Bedeutung der Diagnostik, auch in Bezug auf Leistungen |
| 11.12 | Akutbedarf nur über Kindeswohlgefährdung oder Ähnliches durch Fachkraft abbildbar |
| 11.13 | Auswirkungen Corona: erhöhter Bedarf |
| 11.14 | Herausforderung: Transition in Erwachsenen-Medizin |
| 11.15 | zu wenige Hilfen für Krankheitsbild |
| 11.16 | (fehlende) Infos, (Wissen um) Beratungsstellen, Vermittlung |
| 11.17 | zu wenig Entlastungsangebote für Familien (psychosozial etc.) |
| 11.18 | System: unklarer Zugang, Zuständigkeiten, Anspruchslage |
| 11.19 | schlechtere/erschwerte Versorgung Familien mit Migrations- oder Fluchtgeschichte |
| 11.20 | gezielte Unterstützung durch Fachkraft in häuslichem Setting nötig |
| 11.21 | Aussagen von Fachkräften haben unterschiedliches Gewicht (Kita versus Ärzt*in) |
| 11.22 | fehlende (qualifizierte) beratende, allgemein helfende Fachkraft |
| **12** | **Folgen einer suboptimalen Patient Journey** |
| 12.1 | verpasste Entwicklungschancen |
| 12.2 | psychische Belastung der Fachkraft |
| 12.3 | akute Gesundheitsgefährdung |
| 12.4 | präventable Komorbiditäten |
| 12.5 | Gefährdung Kindeswohl |
| 12.6 | psych Auffälligkeiten, Erkrankungen |
| 12.7 | Entwicklung gesundheitsschädlicher Verhaltensweisen |
| 12.8 | schlechteres Wohlbefinden, Mobbing |
| 12.9 | soziales Missverhalten |
| 12.10 | Kind wird missverstanden |
| 12.11 | Eingriffe in die Familie |
| 12.12 | unwürdige Lebensumstände |
| 12.13 | beengter, unfreier Lebensraum |
| 12.14 | misslingende Lebensführung |
| 12.15 | schlechterer Schul-& Lebensverlauf |
| **13** | **gewünschte Anlaufstellen für Familien** |
| 13.1 | neuer Wunsch/Vorschlag gewünschte Anlaufstellen |
| 13.2 | Gespräch bei Arzt mit weiterem Plan, Anlaufstellen |
| 13.3 | zentrale, offiziell organisierte Anlaufstelle/Beratung |
| 13.4 | Kinderarztpraxis als zentrale Anlaufstelle gewünscht |
| 13.5 | Vermittlung an andere Betroffene |
| 13.6 | mehr Wissen, Beratung, Vermittlung bei Krankenkassen |
| **14** | **Lösungsideen & Wünsche** |
| 14.1 | neuer Wunsch/Vorschlag Lösungsideen, Wünsche |
| 14.2 | Sozialarbeiter:innen für Kinderarztpraxen |
| 14.3 | Ausbau Digitalisierung |
| 14.4 | Infoangebote für Fachkräfte über Leistungen, Zuständigkeiten |
| 14.5 | mehr individualisierte Therapie, Anpassung an Kind |
| 14.6 | ortsungebundene Online Leistungen erstattungsfähig durch Krankenkasse |
| 14.7 | mehr ambulante statt vollstationäre Angebote |
| 14.8 | Reduktion von Wartezeiten durch verschiedene Maßnahmen |
| 14.9 | Aufklärung der Fachkraft über Krankheitsbilder |
| 14.10 | Aufklärung der Bevölkerung über Krankheitsbilder |
| 14.11 | Fortbildungen, aktuelleres Fachwissen bei Fachkräften |
| 14.12 | Ausbau von Strukturen für Kinder mit chronischen Erkrankungen |
| 14.13 | aufsuchende Hilfen |
| 14.14 | direkte Unterstützungsperson für Familie, die entlastet |
| 14.15 | Abgestufte Unterstützungsangebote für Familien mit Problemen |
| 14.16 | Austauschangebote für Familien mit präventivem Ansatz |
| 14.17 | zentrale Ablauf-, Vermittlungs-, Infostelle für Familien |
| 14.18 | mehr (ausgebildete) Fachkräfte & Institutionen |
| 14.19 | bessere grundlegende Finanzierung, Leistungsbewilligung |
| 14.20 | spezialisierte Ansprechpartner |
| **15** | **Messgrößen Evaluation Patient Journey** |
| 15.1 | neuer Wunsch/Vorschlag Messgrößen Evaluation |
| 15.2 | Erreichbarkeit der Fachkraft für Familien, Kommunikation |
| 15.3 | Umsetzung der Fachkraft-Empfehlungen durch Eltern |
| 15.4 | Umversorgung bei Fachkraft Wechsel |
| 15.5 | Bewilligungspraxis Versorgungsleistungen (Zeiten, Ablehnungen) |
| 15.6 | Dauer zwischen Diagnose/Therapie oder Empfehlung & Start der Leistung, Wartezeit auf Termine |
| 15.7 | krankheitsspezifische Outcomes, Verläufe |
| 15.8 | Integration ins Schulsystem |
| 15.9 | abhängig von Lage/Bereitschaft/Präferenzen der Familie |
| 15.10 | Code in ICD für Einschätzung der Versorgung durch die Kinderarztpraxis |
| 15.11 | Zeitaufwand der Fachkraft für Betreuung des Patient*in |
| 15.12 | Informationsstand von Eltern/Kindern zu Erkrankung, Versorgungssystem |
| 15.13 | Anzahl der Fachkraft Kontakte |
| 15.14 | Versorgungskontakt zwischen Pflichtterminen |
| 15.15 | Anzahl verschiedener Anlaufstellen bis Diagnose gesichert ist |
| 15.16 | Anzahl stationärer Aufenthalte |
| 15.17 | Anzahl notfälliger Vorstellungen aufgrund der Erkrankung |
| 15.18 | Verfügbarkeit von Familienbegleiter*innen |
| 15.19 | Umfang elterlichen Engagements, das nötig für gute Versorgung |
| 15.20 | fühlen Eltern sich gut aufgehoben, ausreichend versorgt |
| 15.21 | Be- bzw. Entlastung der Familie |
| 15.22 | Teilhabe, real möglich, (selbstständige) Freizeitgestaltung |
| **16** | **potentiell vermeidbare Aufwände, Belastungen** |
| 16.1 | hoher Aufwand Bürokratie |
| 16.2 | mehrfache Diagnostik, Untersuchungen & Gründe dafür |
| 16.3 | potentiell unnötige diagnostische/therapeutische Verfahren, Gründe hierfür |
| 16.4 | Überschneidung von Leistungen in parallelen Angeboten |
| 16.5 | unnötige Ablehnungen, Gutachten |
| 16.6 | (unnötiger) Wunsch der Familien nach spezialisierter Behandlung |
| 16.7 | Familien wollen potentiell unnötige Leistungen, Hilfsmittel |
| **17** | **Lebenswelt Kita, Schule** |
| 17.1 | Therapieangebote in Schule, Kita |
| 17.2 | schnelle Rückkehr in Regelalltag/-schule |
| 17.3 | Probleme in der Schule/andere Instanz lehnt das Kind ab |
| 17.4 | fehlende (auch personelle) Ausstattung Schule, Kita |
| 17.5 | hilfreich: flexiblere Schulbesuchsformen, z.B. online |
| 17.6 | Schulausschluss |
| 17.7 | zu wenig I-Helfer, abgelehnte Anträge |
| 17.8 | (fehlendes) Wissen zu Krankheitsbild Erzieher*innen,Lehrer*innen |
| 17.9 | Schwierigkeiten des Kindes bei Konzentration, oder im Verhalten |
| 17.10 | Unterstützung beim Therapiemanagement in der Schule nötig |
| 17.11 | unzureichender Personalschlüssel |
| 17.12 | Unterschiede zwischen Bundesländern: Schwerpunkte Kita und Schulformen |
| 17.13 | Beschreibung des Schulalltags |
| 17.14 | Kita, Schule als einziger Lebensraum außerhalb des Zuhauses für Kind |
| **18** | **ideale Patient Journey** |
| 18.1 | neuer Wunsch/Vorschlag für ideale Patient Journey |
| 18.2 | reibungslose Leistungsbewilligung |
| 18.3 | mehr interdisziplinärer Austausch, intersektorale Zusammenarbeit |
| 18.4 | Erkennen von Auffälligkeiten, Einleitung einer Diagnostik durch Fachkraft |
| 18.5 | zentrale Anlaufstelle |
| 18.6 | schnelle (vorläufige) Diagnostik, ausreichende Kapazität des Versorgungsystems |
| 18.7 | zentrale/gute Informationsquelle für Familien, gute Kommunikation |
| 18.8 | Familien Selbstwirksamkeit vermitteln |
| 18.9 | auf Bewältigung durch Familien achten, ggf. aufsuchend helfen |
| 18.10 | regelhafter Kontakt mit Sozialberatung oder psychologischem Dienst |
| 18.11 | schnelle & engmaschigere Anbindung an Therapie & Support-System |
| 18.12 | Fallmanager*in |
| **19** | **gesellschaftliche Vorurteile** |
| 19.1 | (keine) fehlende gesellschaftliche Akzeptanz, Aufklärung, Anpassung |
| 19.2 | Vorurteile & Klischees |
| 19.3 | Eltern wird Schuld an Verhalten des Kindes gegeben |
| 19.4 | Fachkräfte unterstellen Eltern, Kind zu vernachlässigen, zumisshandeln |
| 19.5 | Diagnose wird angezweifelt |
| 19.6 | Testmanuals zu wenig divers |
| 19.7 | hoher Bekanntheitsgrad, geringe Berührungsängste mit Erkrankung |
| **20** | **Teilhabe** |
| 20.1 | nicht erreicht |
| 20.2 | Teilhabe funktioniert gut |
| 20.3 | Selbstständigkeit des Kindes eingeschränkt |
| 20.4 | Teilhabe bei gutem Therapiemanagement prinzipiell uneingeschränkt möglich |
| 20.5 | Teilnahme Klassenfahrt, (Schul)ausflug |
| 20.6 | Erfahrung: Erkrankung ist gut kontrollierbar |
| 20.7 | (Therapie) Ziele bzgl. Teilhabe |
| 20.8 | eingeschränkte soziale Kontakte |
| 20.9 | Unterstützung des Kindes durch Peers |
| 20.10 | Kind will nicht durch Erkrankung unter Peers auffallen |
| 20.11 | (fehlende) Leistungen zur Unterstützung von Teilhabe |
| 20.12 | starke Einschränkung des Kindes im Alltag |
| 20.13 | fehlende Finanzierung zur Unterstützung von Teilhabe |
| 20.14 | eigenständiges Therapiemanagement durch Kind, Auswirkungen auf Teilhabe |
| 20.15 | fehlende Aufklärung über Erkrankung im Freizeitbereich |
| 20.16 | Angebote/Betreuer wollen mehr Infos & Sicherheit |
| 20.17 | Ängste bzgl Sicherheit schränken ein |
| 20.18 | Strukturen in Schule/Kita erschweren Teilhabe |
| 20.19 | konkrete Verbesserungsvorschläge zur Verbesserung Teilhabe |
| 20.20 | Mobbing |
| 20.21 | gute Verarbeitung der Erkrankung durch das Kind |
| 20.22 | I-Helfer: Sicherheit versus Selbstständigkeit |
| 20.23 | fehlende Freizeit/Ferienangebote für Kind |
| **21** | **Thema Fachkraft Verfügbarkeit, finanzielle Ressourcen** |
| 21.1 | fehlende Zeit für interdisziplinären Austausch, Kommunikation |
| 21.2 | Ressourcen fehlen, um nach aktuellstem wissenschaftlichem Stand zu therapieren |
| 21.3 | fehlende Strukturen zur Lösung von Versorgungsproblemen Alltag |
| 21.4 | fehlende Zeit der Fachkraft für weitere/bessere Behandlung |
| 21.5 | keine zu Krankheitsbild spezialisierte Fachkraft verfügbar |
| 21.6 | Entlassung nach Erstdiagnose bevor Eltern und Kind sicher im Therapiemanagement sind |
| 21.7 | (fehlende) regionale Versorgungsstrukturen |
| 21.8 | lange Wartezeiten für Diagnostik, Therapie, da zu wenige Fachkräfte |
| 21.9 | Schließung von Einrichtungen verschärft Versorgungsengpass |
| 21.10 | Kostendruck der Krankenkassen führt zu Ablehnung von Anträgen |
| 21.11 | gute Verfügbarkeit von Fachkräften |
| 21.12 | lange Wartezeiten, zu geringe Kapazität |
| 21.13 | unzureichende Versorgung durch Fachkraft-Mangel |
| 21.14 | unzureichende Kapazität Kinderarztpraxis für Bedarfe von Kindern mit chronischen Erkrankungen |
| **22** | **familiäre Belastung in Zusammenhang mit Versorgung** |
| 22.1 | große Entfernungen, Fahrtzeit |
| 22.2 | Stellenwert elterlichen Engagements & Ressourcen für gute Versorgung |
| 22.3 | Unterstützung für Familien über Erstdiagnose hinaus nötig |
| 22.4 | Belastung durch Ablehnung von Anträgen, Prüfungen durch MDK |
| 22.5 | Belastung durch beengte Wohnverhältnisse |
| 22.6 | Organisations-, Koordinationsaufwand bei Familien |
| 22.7 | wiederholte Krankenhaus Aufenthalte wegen Komplikationen |
| 22.8 | familiäre Belastungen führen zu schlechterem Therapiemanagement |
| 22.9 | Vereinbarkeit von Care Arbeit und Erwerbstätigkeit |
| 22.10 | "richtige" Einordnung des Entwicklungsstandes des Kindes durch die Eltern |
| 22.11 | Zeitaufwand: Therapiemanagement in der Schule |
| 22.12 | Zeitaufwände: Begleitung zu Schulausflug, Klassenfahrt |
| 22.13 | finanzielle Notlagen, Schwierigkeiten |
| **23** | **Familiensystem** |
| 23.1 | gesundheitliche Belastung der Eltern |
| 23.2 | (fehlende) Entlastung, psychosoziale Unterstützung |
| 23.3 | psychische Belastung des Kindes |
| 23.4 | psychische Belastung der Familien |
| 23.5 | Einfluss von Scham |
| 23.6 | weniger schwerwiegende Erkrankung wird auf die leichte Schulter genommen |
| 23.7 | vielfältige Gründe für (nicht) gelingende Anpassung an Situation |
| 23.8 | Management durch Eltern: Erkrankung gut im Griff |
| 23.9 | Alltag: es müssen Dinge beachtet werden |
| 23.10 | Erleichterung durch feststehende Diagnose |
| 23.11 | (keine) Schwierigkeiten mit Krankheitsakzeptanz |
| 23.12 | Veränderung der Lebensumstände beeinflusst Umgang mit Erkrankung des Kindes |
| 23.13 | besondere Belastung, wenn Geschwisterkind ebenfalls nicht gesund |
| 23.14 | Probleme im Alltag für Kind |
| 23.15 | Erkrankung des Kindes wird von Eltern als Störfaktor empfunden |
| 23.16 | Trennung der Eltern oder Erkrankung eines Elternteils führt zu Wegfall einer Betreuungsperson |
| 23.17 | v.a. ein Elternteil übernimmt Krankheitsmanagement |
| 23.18 | Schwierigkeiten Betroffener aufgrund der Erkrankung |
| 23.19 | Aushandlungsprozesse, Konflikte zwischen Eltern und Kind zu Erkrankung und deren Management |
| 23.20 | Unsicherheit der Eltern im Umgang mit der Erkrankung & dem Kind |
| 23.21 | Sorge der Eltern bzgl. Verlauf der Erkrankung, Lebensperspektive |
