# Supplementary material for: “It cannot depend solely on the parents”—professionals’ perspectives on patient journeys of children with chronic conditions across health and social care in Germany
Source: Eur J Pediatr. 2026 Oct 22;185(11):872. doi: 10.1007/s00431-026-07533-x (PMC13687312; doi:10.1007/s00431-026-07533-x)
Supplement: Supplementary file 4 [file 431_2026_7533_MOESM4_ESM.docx]

Introduction:

Thank you very much for agreeing to participate in our study.

In the PICAR project, we are interested in so-called patient journeys — by this, we mean all interactions and contacts that patients have with the healthcare system. As part of the project, we are conducting an interview study with children, parents, and healthcare professionals to explore, among other things, what determines the quality of care from their perspective and which processes lead to burdens, additional effort, or extra costs. The findings from these interviews will be further explored in subsequent sub-studies within the project, with the aim of developing recommendations for better coordinated, or “integrated,” care for children with chronic illnesses.

As a healthcare professional involved in the care and support of children with chronic conditions or disabilities and their families, you have valuable insights into everyday healthcare provision. I would like to learn more about your experiences and perspectives in today’s conversation.

Our conversation today will take approximately one hour. You are free to skip any question or end the interview at any time. Everything we discuss will remain confidential. With your consent, the interview will be audio-recorded, but it will not be linked to your name. After the interview has been transcribed, the audio recording will be deleted.

Do you have any questions before we begin?

**Definition Patient Journey**: Definition Patient Journey: all encounters and contacts that patients have with the healthcare system – includes the medical domain, but also care structures beyond that in the home or school environment.

Timeframe: first symptoms/signs – diagnosis – initiation of treatment – ongoing care over the life course (childhood and adolescence)

| **Introduction: Professional Background and Role** | |
| --- | --- |
| **Guiding Question** | **Probing questions** |
| What is your occupation? | - How many years have you been in this field? - Do you specialize in certain areas in your work? |
| Can you give an example of a typical medical condition that you frequently care for here? | - Which care services do you typically provide in these cases? - How are patients referred or directed to you? - How large is the catchment area from which you care for patients? |
| **Experiences with Patient Journeys** | |
| **Guiding Question** | **Probing questions** |
| Using the typical condition you just described, can you explain, based on your experience, how the patient journey of such a patient usually unfolds? | - Can you describe the timing and sequence of events between the different stops of the patient journey? - Which actors and institutions are you in contact with when caring for your patients? - How is communication and exchange organized with the actors you mentioned? - Have you encountered situations where examinations were conducted or services provided that, in your view, were not necessary? If yes, why? - How long do patients typically wait for an appointment with you? In your view, how could waiting times be reduced? - Where do you get your information about other care services available for your patient population, and to which providers can you refer them? What would you consider an appropriate source of such information? - Do you have the impression that the design of a typical patient journey enables patients to participate socially (e.g., in society, education, etc.)? - Optional: To what extent are patients usually involved in decisions regarding further treatment planning? |

| **Positive Aspects of Care Provision** | |
| --- | --- |
| **Guiding Question** | **Probing questions** |
| In your view, what aspects of medical care for your patients already work well? | - Why? - What are the most important elements or factors that make care work well? - What support would be needed to maintain or further develop these well-functioning structures? |
| **Challenges in Care Provision** | |
| **Guiding Question** | **Probing questions** |
| In your view, what aspects of medical care for your patients need improvement? | - Why? - What are the main challenges to providing adequate care for your patients? - Do the difficulties you described lead to additional effort or costs for you or your institution? - Where do you see particular burdens for your patients and their families in medical care?   Possible follow-up topics:   - Do the difficulties you described affect your relationship with your patients? - Do these factors make it harder for your patients to access medical care? |

| What factors or metrics do you think one should look at to assess the quality of such a patient journey? | - Can you provide examples? - Is there a metric related to health insurance billing that could indicate how successful a patient journey is? - What would one need to ask healthcare professionals to assess the quality of a patient journey? - What would one need to ask parents and children? |
| --- | --- |

**Final Section – Summarizing and Prioritizing**

| **Guiding Question** | **Probing questions** |
| --- | --- |

| To summarize, what would an ideal patient journey for a child look like, from diagnosis to long-term care? | - Can you describe this using an example condition? |
| --- | --- |

Is there anything we haven’t discussed that you think is important on this topic?
