# Supplementary material for: “It cannot depend solely on the parents”—professionals’ perspectives on patient journeys of children with chronic conditions across health and social care in Germany
Source: Eur J Pediatr. 2026 Oct 22;185(11):872. doi: 10.1007/s00431-026-07533-x (PMC13687312; doi:10.1007/s00431-026-07533-x)
Supplement: Supplementary file 5 [file 431_2026_7533_MOESM5_ESM.docx]

# Supplement

Supplement table 1: Cited Participant Quotes in Original German and English Translation.

|  | **English Translation** | **Original German** |
| --- | --- | --- |
| P 12, §49 | The coordination of measures is oftentimes unsatisfactory. […] If you take all that into account, then you often have very different worlds existing in parallel, which then have the child as their intersection, so to speak. But it is then very difficult to bring the different spheres together or to link them in the interests of the child. So coordination across sectors or structures is incredibly cumbersome. | Die Koordination der Maßnahmen ist häufig unbefriedigend. […] Wenn man das alles noch mit reinnimmt, dann sind das ja häufig ganz unterschiedliche, parallel existierende Welten, die dann als Schnittmenge sozusagen das Kind haben. Aber es dann ganz schwierig ist, die unterschiedlichen Bereiche zusammenzubringen oder im Sinne des Kindes zu verknüpfen. Also eine Sektoren- oder Strukturen- übergreifende Koordination ist unheimlich schwerfällig. |
| P 8, §36 | The child moves back and forth between these individual areas. However, it is not possible to establish a common thread for the child unless everyone works together and is connected. | Das Kind wechselt zwischen diesen einzelnen Bereichen hin und her. Und es kann aber kein roter Faden entstehen für das Kind, wenn nicht alle miteinander zusammenarbeiten und vernetzt sind. |
| P 15, §73 | Because there is no coordination, or because no concrete goals are defined. So I believe that due to this lack of clear goal-setting, energy is often wasted. | Weil sich nicht abgesprochen wird oder weil keine konkrete Zielsetzung gemacht wird. Also da glaube ich, dass durch diese fehlende konkrete Zielsetzung oft ja Energie verpufft. |
| P 10, §63 | So, data protection really does hinder communication. | Also der Datenschutz, der behindert dann schon sehr in der Kommunikation. |
| P 11, §33 | And then it goes back and forth like a ping-pong ball. | Und dann geht das wie so ein Pingpong-Ball. |
| P 11, §33 | It’s just that the schedule is so tightly packed that in practice […] you always feel like you’re on the front line. | Das ist halt wirklich so hart getaktet, die Zeit, dass man in der Praxis […] immer, wie an der Front ist. |
| P 9, §49 | For me, the provision of care for children and young people always requires taking into account their age and stage of life. | Die Versorgung von Kindern, von Jugendlichen ist für mich immer eine Versorgung mit dem Blick des Lebensalters, mit der Lebensphase. |
| P 12, §35 | posing a risk to the children’s development | entwicklungsgefährdend für die Kinder |
| P 8, §50 | that this is actually a legal right for these children | dass das eigentlich dann ein Recht ist für diese Kinder |
| P 19, §55 | I fear that some children will not receive the help they need when they need it. And that this will have long-term consequences. | Ich befürchte, dass einige Kinder nicht die Hilfe kriegen, zu dem Zeitpunkt, wo sie es bräuchten. Und sich dementsprechend Langzeitfolgen ergeben. |
| P 8, §40 | It cannot depend solely on the parents, so to speak, whether they are able to obtain all this information themselves or not. | das kann nicht sozusagen nur alleine von den Eltern abhängen, ob sie in der Lage sind, sich diese Informationen alle selber zu beschaffen oder nicht. |
| P 13, §99 | “Parents who, yes, have an academic degree […] often have other options available to them, including financial resources, […] to navigate the bureaucratic jungle. But I would say that about a third of our students do not have this luxury, so to speak.” | „Eltern, die, ja, einen akademischen Abschluss haben, […] die haben oftmals dann noch andere Möglichkeiten, auch zum Teil finanzieller Art, […] sich durch den Behördendschungel zu kämpfen. Aber ich würde mal sagen, ein Drittel unserer Schüler hat diesen Luxus, in Anführungszeichen, nicht.“ |
| P 5, §59 | very, very low threshold, which is extremely good | sehr, sehr niedrigschwellig und das ist extrem gut |
